# Event-Related Potential Assessment of Visual Perception Abnormality in Patients With Obstructive Sleep Apnea: A Preliminary Study

**DOI:** 10.3389/fnhum.2022.895826

**Published:** 2022-06-30

**Authors:** Chao Yang, Changming Wang, Xuanyu Chen, Bing Xiao, Na Fu, Bo Ren, Yi Liu

**Affiliations:** ^1^College of Psychology and Mental Health, North China University of Science and Technology, Tangshan, China; ^2^Department of Respiratory Medicine, Civil Aviation General Hospital, Beijing, China; ^3^Department of Neurosurgery, National Center for Neurological Disorders, Xuanwu Hospital, Capital Medical University, Beijing, China; ^4^Personality Development and Social Adjustment Lab, Faculty of Psychology, Southwest University, Chongqing, China

**Keywords:** OSA, visual perception, occipital lobe, ERP, PSG

## Abstract

This study investigated the effect of obstructive sleep apnea (OSA) on the neural mechanism of visual perception. A preliminary case-control study was conducted. Seventeen patients with moderate to severe OSA in the sleep center of Civil Aviation General Hospital and 20 healthy controls matched for age, sex, and education were recruited. The participants accepted the perceptual contour integration task, compared the differences in behavioral indicators between the two groups, and compared the differences in electroencephalography (EEG) data between the two groups through event-related potential (ERP) technology. The groups did not differ significantly in age and gender, but they differed significantly in body mass index (BMI) (OSA higher than healthy controls, *p* < 0.05). The groups were not statistically different in terms of sleep structure and total sleep time. AHI, sleep efficiency, and minimal SaO_2_ value in the OSA group were significantly different from those of the control group. The results showed that the average reaction time of the OSA group was significantly longer than that of the healthy control group in the contour integration task. There was no significant difference in the accuracy rate. The results of EEG showed that the amplitudes of N100 of the OSA group were significantly higher than those of the control group at O1, Oz, and O2 electrodes. There was no significant difference in latency between the two groups. At the FCz electrode, the amplitudes of N200 of the OSA group were significantly higher than those of the control group. Therefore, we concluded that in the early stage of the perceptual integration task, although the neural response activity of patients with moderate and severe OSA was not accelerated, they need to call on more psychological resources, activate more neurons in the contour global recognition processing stage, and the compensatory effect of frontal lobe appeared in the stage of visual perception.

## Introduction

Obstructive sleep apnea (OSA) is a worldwide common disease characterized by recurrent partial or total obstruction of the upper respiratory tract during sleep, and these respiratory events lead to frequent sleep arousal and intermittent hypoxemia (Benjafield et al., 2019). OSA is a significant public health concern, and an estimation of the global prevalence and burden of obstructive sleep apnea from a reliable literature-based analysis indicated that 936 million adults aged 30–69 years had mild to severe obstructive sleep apnea and 425 million adults aged 30–69 years have moderate to severe obstructive sleep apnea globally (Benjafield et al., 2019). It is also associated with an increased risk of several coexisting conditions, such as cardiovascular disease, stroke, and diabetes (Veasey and Rosen, 2019). Moreover, it is associated with an increased risk of developing cognitive decline in attention, working memory, and executive function (Liguori et al., 2020), which is closely related to the structural and functional changes in the prefrontal cortex and hippocampus.

Previous studies have found that patients with severe OSA have poor global cognition, delayed recall, and attention performance. This indicates that severe OSA may be an independent factor affecting these three factors (Andreou et al., 2014). Other studies suggest that patients with OSA have worse executive function than healthy people (Li et al., 2016). However, the above studies focus on the higher cognitive function, but the visual image from seeing to recognition needs to go through a fixed time process; before the completion of recognition, it is bound to go through the process of feeling and perception.

It is well known that the occipital lobe is responsible for visual perception of information and operational storage. It receives incoming information, which is processed and immediately sent to the hippocampus, where it is formed into memory. However, less is known about alterations of the occipital lobe in OSA. Several studies (Bart and Sangal, 1997; Ejma et al., 2005; Liguori et al., 2018) reported that patients with OSA presented prolonged visual evoked potential alteration, and one study (Liguori et al., 2018) confirmed that CPAP therapy could significantly improve VEP. Giora observed an overall perceptual deficit consisting in OSA patients invested by means of a visual search task (Giora et al., 2017). Lusic confirmed that individauls with severe OSA had impaired speed of perception, convergent, and operative thinking (Kalcina et al., 2020).

It is important to question whether the impairment of higher cognitive function in patients with OSA is partly due to the impairment of visual perceptual processes. When part of the visual image is visible (such as occlusion and light difference), the brain will go through a neural process to fill in the missing information so as to achieve recognition (Doniger et al., 2001). When we saw ambiguity or incomplete visual images, brain areas in the occipital, temporal, and parietal lobes participate in the integration process. In fMRI studies, lateral occipital complex (LOC) was found to be more activated by ambiguity stimuli than clarity stimuli in the visual perception task (Hegdé et al., 2008; Freud et al., 2018).

Electroencephalography (EEG) has higher temporal resolution than functional magnetic resonance imaging (fMRI), so this technique can well describe the temporal process of sensory and perceptual processes in the formation of incomplete vision. Event-related potential (ERP) is a kind of special brain evoked potential, which acts on the sensory system or a certain part of the brain through a specific stimulus. When the stimulus is given or withdrawn, the potential changes in the brain area are caused. It reflects the changes of the brain neuro-electrophysiological in the cognitive process. In ERP studies, N100 is a typical component related to visual sensory and perceptual processing (Luck and Kappenman, 2013). It is believed to reflect the early sensory process of low-level visual information. N100 is a component that begins at 130 ms after the presentation of visual objects and peaks at 160–180 ms, which can be observed at electrodes around bilateral occipital regions (Itier and Taylor, 2004; Dai et al., 2022). N200 reflects general executive and cognitive functions, such as stimulus recognition, and peaks at 200–300 ms, which can be observed at electrodes around frontal central regions.

In summary, this preliminary study adopted electrophysiological indicators that effectively characterized individual visual perception and cognitive function to assess whether moderate to patients with severe OSA show worse performance on visual perception than healthy control people. To evoke those ERP components, we used the contour integration task with a typical visual perception integration process. Therefore, this study investigates (1) whether the visual perception ability reflected by the N100 of patients with OSA was abnormal when they completed the task; (2) whether the cognitive function reflected by the N200 of patients with OSA needs the frontal lobe to compensate for the loss of the visual perception.

## Materials and Methods

### Participants

This study was a preliminary case-control study conducted at the Civil Aviation General Hospital. It was approved by the Ethical Committee of Civil Aviation General Hospital (Batch number: 201801). The participants (patients with OSA or healthy control people) were either recruited through those attending sleep clinics for evaluation of possible OSA or from routine physical examination clinics. Inclusion criteria are as follows: (1) adults aged between 18 and 60 years; (2) education years ≥9 years; and (3) first clinic visit and no OSA-related treatment. Exclusive criteria are so followed: (1) any genetic syndrome associated with cognitive disabilities or any chronic or psychiatric condition; (2) use of psychotropic or sedative medicine affecting memory or sleep; and (3) history of central nervous system disease or neuromuscular disease; (4) patients with acute upper respiratory tract infection or severe heart and lung diseases; (5) suffering from malignant tumors or autoimmune diseases; (6) patient with severe disturbance during polysomnography (PSG) measurement; (7) alcohol or substance abuse/dependence; severe visual and hearing impairment; and recent unexpected major life events (such as unemployment, death of relatives, and major economic losses).

In total, seventeen adults with moderate-to-severe OSA (apnea-hypopnea index, AHI ≥ 15) without prior positive airway pressure treatment (OSA group) and twenty healthy controls (HC group) were identified from December 2020 to January 2022. The sample size was calculated using G-power software. We selected ‘Means: Difference between two independent means (two groups)’ for the ‘Statistical test’ option, ‘A prior: Compute required sample size – given α, power, and effect size’ for the ‘Type of power analysis’ option. Input parameters included: two-tailed test, effect size *d* = 0.95, α err prob = 0.05, power (1-β err prob) = 0.8, allocation ratio N2 (healthy control group)/N1(OSA group) = 1.2. In addition, the output parameters included: sample size group 1 = 17 and sample size group 2 = 20. ERPs were used in this research, PSG data were collected overnight, and the sample size was less than some scale studies or simple behavioral studies, so we defined it as a preliminary study. All participants completed questionnaires during the baseline visit by a study physician, which included a collection of data on demographics, medical history, family history, and education. Body mass index (BMI) was calculated from height and body weight.

### Polysomnography

The PSG device is a Siesta 32 channel wireless telemetry PSG system from Compumedics, Australia. The recording started at 22:00 on the monitoring day and ended between 6:00 and 7:00 on the next day. Sleep monitoring time >7 h, which included electroencephalogram, electrooculogram, electrocardiogram signals, snoring intensity, nasal pressure (nasal cannula), nasal/oral thermistor, thoracic and abdominal movement (inductance plethysmography bands), and oxygen saturation (pulse oximetry). Caffeine, alcohol, and stimulant or sedative drinks or drugs were not permitted before PSG monitoring. According to the guidelines for the Diagnosis and Treatment of Obstructive Sleep Apnea (Revised Edition 2011) issued by the Chinese Medical Association, sleep apnea severity was rated in accordance with the individual’s apnea-hypopnea index (AHI). It was defined as the total number of apneas and hypopneas per hour of sleep and oxygen desaturation (minimal SaO_2_ value) per hour of sleep, where an AHI of 5–15 is designated mild, 16–30 moderate, and >30 events per hour designated as severe OSA.

### Procedure and Electroencephalography Recording

This visual perception research adopts E-prime 2.0 software to program. The experiment includes three blocks in total. Each difficulty grade (Jitter30, Jitter60, and Jitter90) has one block, and each block has sixty trials. The figure outline direction is divided into left and right, and the probability of left and right is 50%. The experiment difficulty setting is based on the MATLAB code programming, and the difference of Jitter30/60/90 lies in the lattice deviation degree and has the gradient difference in the visual perception. The difficulty types include 30-Simple; 60-Moderate; and 90-Difficult.

**FIGURE 1:**
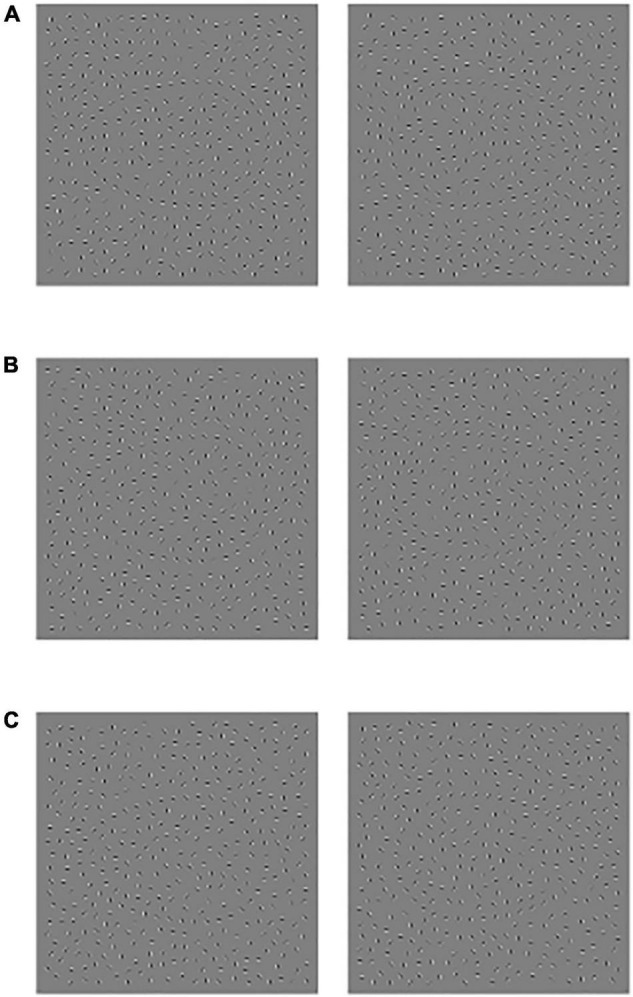
Schematic diagram of experimental material, **(A)** Jitter30, **(B)** Jitter60, and **(C)** Jitter90, the profile orientation of the left side figure is left, and the profile orientation of the right-side figure is right.

**FIGURE 2:**
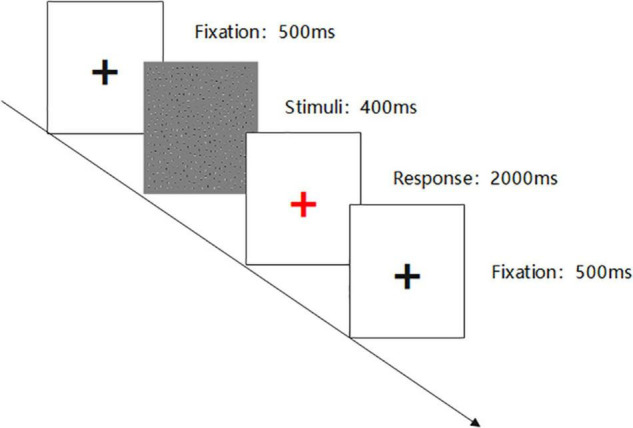
Contour integration experimental flowchart.

Each stimulus presentation time is 400 ms. After the end of the stimulus presentation, the center of the screen will show a red ‘+’ fixation (2,000 ms) to prompt the subjects to respond, requiring the subjects to quickly and accurately answer the contour direction of the figure. To facilitate the subjects to understand the experimental process, the subjects need to pass the practice phase test before the experiment begins. The experimental materials and process are shown in Figures 1, 2.

Electroencephalography signals were recorded using a Neuroscan device using a 64-conductor cap according to the International 10–20 System Extension. The resistance between each electrode and the scalp is controlled to be less than 5 KΩ. For continuous recording, the filter bandpass is 0.05–100 Hz, and the sampling rate is 1,000 Hz. During recording, the Ref electrode is used as the reference electrode, and the electrode between FPz and Fz is used as the ground electrode.

The EEGLAB toolbox in MATLAB is used for the offline processing of EEG data. The preprocessing operation steps are as follows: (1) deleting useless electrodes; (2) reducing sampling rate to 250 Hz; (3) that reference is converted into a whole-brain average reference; (4) off-line filtering, wherein the filtering range is 0.1–30 Hz; (5) carrying out interpolation replacement on the interfered leads in the inspection data; (6) Independent Component Analysis (ICA), to screen and remove artifacts, such as blinking, horizontal EOG, EMG, and ECG, and to avoid their impact on the subsequent data analysis.

The data segmentation was set to −200 ms–800 ms with the stimulus as the time point, and 200 ms before the stimulus as the baseline. The N100 component of the O1, Oz, and O2 electrodes and the N200 component of the FCz electrode were analyzed. The time window of each component is selected as N100 (120–180 ms) and N200 (200–300 ms).

### Statistical Analysis

SPSS 26.0 statistical software was used for data analysis for general information and clinical data. Quantitative data with normal distribution and homogeneity of variance were expressed by M ± SD, and a comparison between groups was performed using the independent sample *t*-test. Behavioral data results were extracted through an E-data file in the E-prime program, including the average reaction time and the accurate rate of subjects with various difficulties; the peak amplitude and peak incubation period of each ERP component were extracted using the EEGLAB toolbox. The demographic data, behavioral accuracy, reaction time, and the amplitude and latency of ERP components at each ERP electrode were analyzed using the independent sample *t*-test between the OSA group and HC group. The difference was statistically significant when *p* < 0.05.

## Results

### Characteristics of the Participants

The baseline characteristics of the participants are shown in Table 1. The groups did not differ significantly in age and gender, whereas they differed significantly in BMI (OSA higher than healthy controls, *p* < 0.05). The groups were not statistically different in terms of sleep structure and total sleep time. AHI, sleep efficiency, and minimal SaO_2_ value in the OSA group were significantly different from those of the control group, *p* < 0.05. No significant difference was found between the two groups in other indexes.

**TABLE 1:** Baseline characteristics of the participants (*M* ± *SD*).

Variables	OSA (*n* = 17)	HC (*n* = 20)	*t*	*p*
**Demographic data**				
Age (year)	42.97 ± 7.15	39.48 ± 8.62	1.325	0.194
Gender (M:F)	13:4	14:6	0.195	0.659
Height (cm)	170.50 ± 7.61	170.47 ± 8.27	0.009	0.992
Weight (kg)	85.59 ± 10.95	73.62 ± 13.44	2.933	<0.05*
BMI (kg/m^2^)	29.69 ± 5.19	25.28 ± 4.04	2.901	<0.05*
**Polysomnographic data**				
Apnea/hypopnea index	44.99 ± 21.93	1.45 ± 1.32	8.174	<0.001**
Apnea index	28.33 ± 13.83	0.35 ± 0.61	8.331	<0.001**
Hypopnea index	16.66 ± 16.27	1.05 ± 1.17	3.946	<0.05*
Minimal SaO_2_ value (%)	73.05 ± 9.52	90.65 ± 2.64	−7.379	<0.001**
Total sleep time (min)	402.4 ± 55.1	395.2 ± 52.4	0.281	0.781
Sleep efficiency (%)	90.78 ± 5.96	84.20 ± 11.48	2.234	<0.05*
Sleep onset latency (min)	16.00 ± 22.21	23.85 ± 30.62	−0.878	0.386
N1 stage proportion (%)	9.25 ± 6.47	6.72 ± 3.88	1.196	0.243
N2 stage proportion (%)	58.72 ± 12.79	57.85 ± 10.54	0.164	0.871
N3 stage proportion (%)	17.55 ± 6.28	22.60 ± 9.58	−1.207	0.239
REM stage proportion (%)	9.45 ± 3.93	12.83 ± 5.71	−1.348	0.190

*p < 0.05, **p < 0.001.

### Behavioral Performance

**FIGURE 3:**
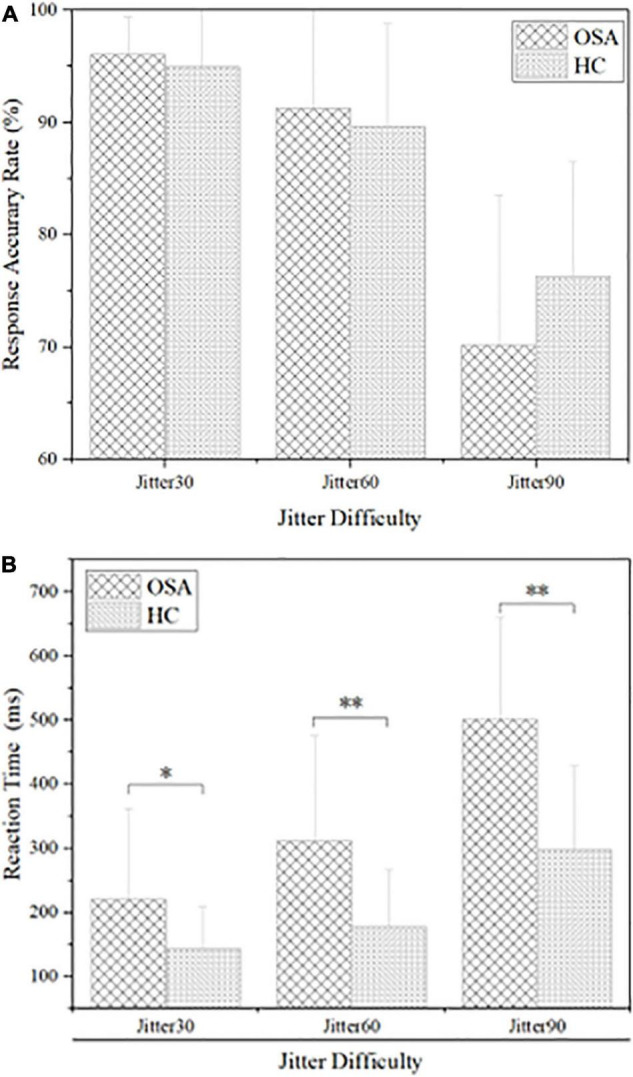
Comparison of behavioral results between obstructive sleep apnea (OSA) group and healthy control (HC) group, **(A)** accuracy rate under each condition; **(B)** reaction time under each condition. *p < 0.05, **p < 0.01.

The results are shown in Figure 3 and Table 2: the average reaction time (RT) of the OSA group was significantly longer than that of the healthy control group under the conditions of Jitter30, 60, and 90, *p* < 0.05. There was no significant difference in the accuracy rate (AR) between the two groups under the conditions of Jitter30, 60, and 90, *p* > 0.05. There was a negative correlation between the minimal SaO_2_ value and the reaction time of Jitter 30, 60, and 90 (*r* = −0.367, *p* = 0.026; *r* = −0.481, *p* = 0.003; *r* = −0.377, *p* = 0.021), that is, the lower the blood oxygen saturation, the slower the behavioral reaction time.

**TABLE 2:** Comparison of behavioral results between obstructive sleep apnea (OSA) group and healthy control group (*M* ± *SD*).

	OSA (*n* = 17)	HC (*n* = 20)	*t*	*p*
Jitter30 AR (%)	96.08 ± 3.28	95.00 ± 7.84	0.529	0.6
Jitter60 AR (%)	91.27 ± 9.02	89.61 ± 9.10	0.537	0.594
Jitter90 AR (%)	70.20 ± 13.39	76.33 ± 10.17	−1.583	0.122
Jitter30 RT (ms)	220.17 ± 142.45	143.05 ± 65.59	2.17	<0.05*
Jitter60 RT (ms)	311.29 ± 163.74	177.78 ± 8.60	3.151	<0.01*
Jitter90 RT (ms)	501.11 ± 259.67	298.75 ± 129.28	3.071	<0.01*

*p < 0.05.

### Event-Related Potential Results

#### N100 Component

**FIGURE 4:**
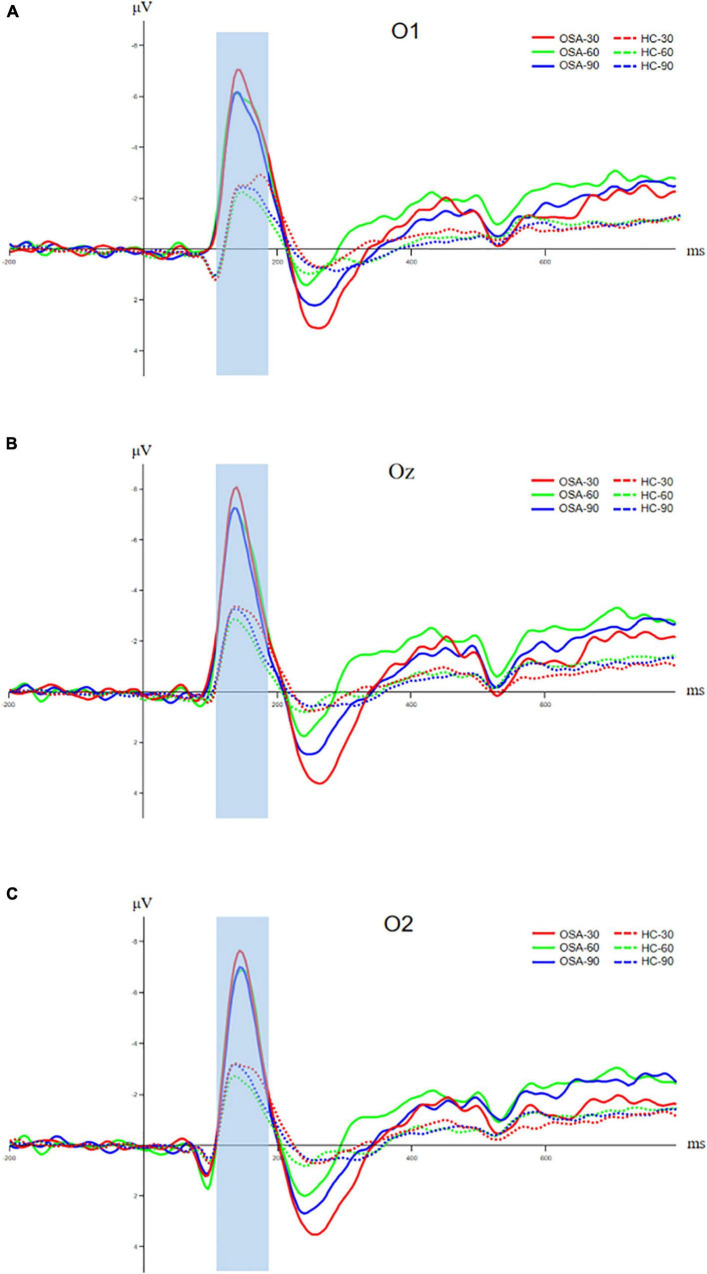
Comparison of N100 components between OSA and healthy control group, **(A)** amplitude and latency of N100 component at O1 electrode; **(B)** amplitude and latency of N100 component at Oz electrode; and **(C)** amplitude and latency of N100 component at O2 electrode. The solid line is the OSA group, and the dotted line is the healthy control group. The red, green, and blue lines represent Jitter30, Jitter60, and Jitter90 conditions, respectively.

The O1, Oz, and O2 electrodes of the occipital lobe region in the back of the head were selected to analyze the N100 component, and the time window was 120–180 ms. The analysis of N100 amplitude and peak latency of the two groups showed that the N1 amplitude of the OSA group was significantly larger than that of the healthy control group at O1, Oz, and O2 electrodes under the three conditions, *p* < 0.05. The peak latency between the two groups in three electrodes has no significant difference, *p* > 0.05. Details are shown in Figure 4 and Table 3.

**TABLE 3:** The amplitude and latency of each event-related potential (ERP) component in the OSA group and healthy control group (*M* ± *SD*).

	Channel	ERP component	OSA (*n* = 17)	HC (*n* = 20)	*t*	*p*
Amplitude (μV)	O1	30-N1	−8.73 ± 5.67	−4.23 ± 2.91	−3.102	<0.01*
		60-N1	−8.32 ± 5.54	−4.12 ± 2.88	−2.956	<0.01*
		90-N1	−8.61 ± 5.50	−3.54 ± 2.75	−3.169	<0.01*
	Oz	30-N1	−10.30 ± 3.88	−5.55 ± 3.92	−3.687	<0.001**
		60-N1	−9.79 ± 4.10	−5.49 ± 3.95	−3.236	<0.01*
		90-N1	−9.99 ± 4.24	−4.74 ± 3.36	−4,199	<0.001**
	O2	30-N1	−9.58 ± 4.53	−5.36 ± 4.03	−2.999	<0.01*
		60-N1	−9.16 ± 4.23	−5.34 ± 3.85	−2.876	<0.01*
		90-N1	−9.20 ± 4.67	−4.59 ± 3.11	−3.575	<0.01*
Latency (ms)	O1	30-N1	144.24 ± 14.66	154.20 ± 19.62	−1.724	0.09
		60-N1	145.88 ± 16.01	145.40 ± 18.27	0.085	0.92
		90-N1	146.12 ± 17.73	144.80 ± 15.11	0.244	0.81
	Oz	30-N1	142.82 ± 14.82	143.60 ± 16.87	−0.147	0.88
		60-N1	145.88 ± 17.04	139.80 ± 14.01	1.192	0.24
		90-N1	145.65 ± 18.44	139.80 ± 14.82	1.069	0.29
	O2	30-N1	141.41 ± 11.48	143.20 ± 16.75	−0.372	0.71
		60-N1	144.24 ± 12.12	139.40 ± 14.11	1.107	0.27
		90-N1	143.77 ± 15.26	139.60 ± 15.07	0.833	0.41

*p < 0.05, **p < 0.001.

#### N200 Component

**FIGURE 5:**
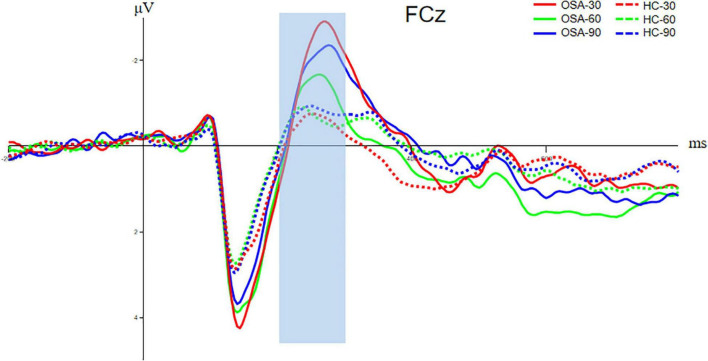
Comparison of N200 components between OSA and healthy control group. The solid line is the OSA group, and the dotted line is the healthy control group. The red, green, and blue lines represent Jitter30, Jitter60, and Jitter90 conditions, respectively.

The FCz electrode in the frontal central area is selected to analyze the N200 component, and the time window is 200–300 ms. The analysis of the N200 amplitude and latency of the two groups indicated that the N200 amplitude of the OSA group was significantly higher than that of the healthy control group under the condition of Jitter30 (OSA: −3.54 ± 2.98 vs. HC: −1.56 ± 2.02, *t* = −2.456, *p* < 0.05). There was no significant difference between Jitter60 and Jitter90 (OSA: −3.16 ± 2.55 vs. HC: −1.82 ± 2.41, *t* = −1.636, *p* > 0.05; OSA: −2.34 ± 2.55 vs. HC: −1.50 ± 1.37, *t* = −1.273, *p* > 0.05). There was no significant difference in the peak latency between the two groups in any of the three conditions, *p* > 0.05, refer to Figure 5 for details. There was a positive correlation between the minimal SaO_2_ value and the amplitude of N200 under the condition of Jitter30 (*r* = 0.356, *p* = 0.03), that is the lower the blood oxygen saturation, the smaller the amplitude of N200.

## Discussion

This study provided significant empirical evidence on the difference between individuals with OSA and healthy control groups in visual perception. Patients with moderate to severe OSA exhibited worse performance in behavioral level and ERP components relative to healthy control people.

The behavioral results of this research indicated longer reaction times for adults with OSA; however, there was no significant difference in accuracy rate between the two groups. This indicates that patients with OSA could accurately recognize the incomplete visual images, but that the time required to process visual information was relatively longer. The ERP results of this research demonstrated that the amplitude of N100 for adults with OSA at O1, Oz, and O2 was greater than in the healthy control group when no differences in peak latency were observed. These findings reflected the fact that the occipital lobe of the adult with OSA required more neural energy when they faced visual stimulation and accomplished sensory-perceptual processing. This might be related to functional decline and lower efficiency of the occipital lobe due to OSA. There is much research that has found a visual perception deficit in patients with OSA, which might suggest a potential risk of OSA-led senior cognitive impairment.

The ERP results of this research also demonstrated that the amplitude of N200 for adults with OSA in besides Jitter 30 was greater than in the healthy control group. There were no significant differences in Jitter 60 and Jitter 90 when peak latency was also not observed among Jitter 30, 60, and 90. N200 can reflect some basic cognitive abilities, such as stimulus recognition. The subjects will experience sensory, perceptual, cognitive, and other stages when they receive visual stimuli in the task. These outcomes suggested compensatory processes in which adults with OSA seize neural resources from the frontal-central (FC) brain region (near FCz) to process the same vestibular information. Patients with OSA have defects in the perceptual processing stage, so they need to call the frontal lobe compensation mechanism to achieve the final recognition of the stimulus. Therefore, we can see that the N200 amplitudes in different conditions were greater in the OSA group than in the control group and also include the difficulty of Jitter60 and 90. The explanation for no difference between Jitter60 and 90 is that Jitter30 is the easiest type, while Jitter60 and 90 are relatively more difficult types. The contour recognition process under the conditions of Jitter60 and 90 is bound to be more complex than that of Jitter30. Therefore, it cannot be ruled out that some OSA cannot accurately identify the stimulus after using frontal lobe compensation due to their age or relatively poor vision. So the amplitude of N200 is smaller, similar to the automatic abandonment at the electrophysiological level.

The minimum SaO_2_ value was correlated with the amplitude of N200 in the Jitter30 condition and the behavioral reaction time in each condition, that is, the lower the blood oxygen saturation, the more severe the nocturnal sleep hypoxia reflected by the minimum oxygen saturation of patients with OSA, the smaller the amplitude of N200 and the slower the behavioral reaction time. The physiological stress response is regulated mainly by the hypothalamus-pituitary-adrenal cortex axis (HPA axis) and sympathetic nervous system axis. Therefore, hypoxia caused by nocturnal apnea affects not only the HPA axis and sympathetic nervous system but also the higher cortex of the brain. This leads to slower behavioral response and an insufficient number or intensity of neurons activated at the cognitive level.

While previous OSA research focused on frontal regions and high-level sentience perception, this research focused on visual perception, as well as the occipital lobe. Neuro-chemical evidence is supportive of white-matter impairment in OSA, particularly in the frontal lobes (Zimmemnan and Aloia, 2006). The NAA/choline ratio was lower in the frontal lobe of patients with OSA (Fu et al., 2016). Nevertheless, previous research mostly focused on foundational perceptions, such as thermic sensation, olfaction, and auditory sensation. The perceptual contour integration task was used in this study, and the visual sensation of participants was also engaged. We believed that this impairment of sensory even perceptual impairment might lead to a compensatory process, which suggests that medical care for OSA is constructive, and even eliminate the predisposing factors of Alzheimer’s disease.

Our findings add to the limited understanding of adults with obstructive sleep apnea in ERP and behavioral experiments, especially in foundational visual perception. But there are still several factors that we need to mention in particular. First, periodic limb movements during sleep (PLMS) also frequently occur in patients with OSA. Since PLMI values during PSG acquisition were not reported in this study, there may be a possibility of OSA comorbidity with PLMS. PLMS is common in OSA and may occur both in close temporal associations with apneas or independently from apneic episodes. But the correlation between PLMS and OSA is not clear. Sleep apnea leads to poor night rest and poor brain function, which affects long-term performance, such as cognitive ability. In patients with significant apnea and elevated PLMS, OSA should be treated prior to assessing the clinical importance of PLMS. Therefore, under the condition of conforming to the rules of the population, we pay more attention to the factors of sleep apnea. Besides, higher PLMS frequency was associated with a greater decline in cognition, particularly in executive function. Considering that human cognitive ability declines with aging and the prevalence of PLMS also increases with age, it is in line with the regularity of neurodegeneration. In addition, dopamine deficiency is also a major factor in PLMS, which affects the prefrontal cortex that regulates executive function. Impaired frontal lobe function can be regarded as the final result at the level of neural electrophysiology, but the cognitive process needs to go through sensation, perception, and advanced cognition. Therefore, the damage to the intermediate process will inevitably affect the final cognitive results. In future studies, we will investigate the differences in cognitive abilities between patients with OSA with and without comorbid PLMS. Finally, due to the small sample size, our results must be interpreted with caution that we defined it as a preliminary study. The current results confirm that patients with OSA have early visual perception impairment. We will include a larger sample size and combine more task paradigms that can reflect visual perception ability in future research to explain the neural mechanism of cognitive impairment in patients with OSA from the perspective of electrophysiology.

Overall, the pattern of results suggests that OSA not only causes symptoms in the respiratory system and sleep but is detrimental to visual perception function. To avoid severe cognitive impairment, early screening and interventions for OSA are urgent. Moreover, the linkage between the occipital lobe and other encephalic regions might have been regarded as a marker for recovery or deterioration of OSA. Visual perceptual ability was introduced into the detection of OSA in this research. N100 and N200 components may become one of the neuroelectrophysiological indexes to evaluate the perceptual ability and cognitive impairment of patients with OSA, which provides a new insight for the clinical diagnosis and evaluation of OSA. Future research needs to consider whether chronic disruption in visual perception function and impairment of nerve function due to OSA will be a predisposing factor to neuronal development and neural degeneration diseases, such as Alzheimer’s disease.

## Data Availability Statement

The raw data supporting the conclusions of this article will be made available by the authors, without undue reservation, to any qualified researcher.

## Ethics Statement

The studies involving human participants were reviewed and approved by Ethical Committee of Civil Aviation General Hospital. The patients/participants provided their written informed consent to participate in this study.

## Author Contributions

CY, NF, and BX recorded the original experimental data. CY and BR analyzed the experiment data. CY, XC, and YL wrote the manuscript. BX completed the ethic files. CW designed the experiments and examined the manuscript. YL was responsible for the revision and supervision of this manuscript. All authors contributed to the article and approved the submitted version.

## Conflict of Interest

The authors declare that the research was conducted in the absence of any commercial or financial relationships that could be construed as a potential conflict of interest.

## Publisher’s Note

All claims expressed in this article are solely those of the authors and do not necessarily represent those of their affiliated organizations, or those of the publisher, the editors and the reviewers. Any product that may be evaluated in this article, or claim that may be made by its manufacturer, is not guaranteed or endorsed by the publisher.
